# Progressive censoring schemes for marshall-olkin pareto distribution with applications: Estimation and prediction

**DOI:** 10.1371/journal.pone.0270750

**Published:** 2022-07-27

**Authors:** R. Alshenawy, Hanan Haj Ahmad, Ali Al-Alwan

**Affiliations:** 1 Department of Mathematics and Statistics, College of Sciences, King Faisal University, Al-Ahsa, Saudi Arabia; 2 Faculty of Commerce, Department of Applied Statistics and Insurance, Mansoura University, Mansoura, Egypt; 3 Department of Basic Science, Preparatory Year Deanship, King Faisal University, Al-Ahsa, Saudi Arabia; University of Science and Technology of China, CHINA

## Abstract

In this paper two prediction methods are used to predict the non-observed (censored) units under progressive Type-II censored samples. The lifetimes of the units follow Marshall-Olkin Pareto distribution. We observe the posterior predictive density of the non-observed units and construct predictive intervals as well. Furthermore, we provide inference on the unknown parameters of the Marshall-Olkin model, so we observe point and interval estimation by using maximum likelihood and Bayesian estimation methods. Bayes estimation methods are obtained under quadratic loss function. EM algorithm is used to obtain numerical values of the Maximum likelihood method and Gibbs and the Monte Carlo Markov chain techniques are utilized for Bayesian calculations. A simulation study is performed to evaluate the performance of the estimators with respect to the mean square errors and the biases. Finally, we find the best prediction method by implementing a real data example under progressive Type-II censoring schemes.

## 1. Introduction

Studying new lifetime models has become necessary and essential as many applications appeared in natural sciences. Over the last four decades, many authors focused their work on generating new lifetime distributions that may fit the experimental data, for example, medical, engineering, social sciences, reliability analysis, and others. In literature, those new models possess good properties and others were superior relative to the original ones. Generalizations of well-known distributions were applied to describe various phenomenal data. One may refer to [1], later [2] and others. The new method which was proposed by Marshall and Olkin was about obtaining a new distribution depending on adding a parameter to the original one. The generated family of distributions is more flexible and has the original distribution as a particular case.

Many physical and lifetime applications were discussed in literature concerning Marshall-Olkin distribution (MO) see for example [3–13].

In this paper, we will consider the Marshall Olkin family with Pareto distribution as a baseline. Marshall-Olkin Pareto (MOP) distribution was studied by [13] under complete sample data. The authors used several estimation methods and found that it has good properties and behaves well as a generalization of the well-known Pareto distribution.

In reliability and lifetime experiments, units under test can be lost or taken out from the experiment before failure. The loss may not be planned, such as in the accidental failure of some units under experiment or if a unit drops out. Sometimes, the experiment must stop due to the unavailability of testing facilities. Most often, the removal of units from an experiment is pre-planned and is made due to cost and time limitations. The benefit of progressive censoring lies in its efficient utilization of the available resources, so when some of the surviving units in the experiment are removed early, they can be used for some other tests. In reliability and life testing experiments, one of the primary purposes is to draw inference about unknown parameters of interest of an underlying lifetime distribution based on certain censored observation, see [14]. Mainly in such studies, either the interest is to provide estimates for unknown parameters or draw some prediction inference about future observations.

The most frequently used censoring schemes are called progressive Type-I and progressive Type-II censoring. One can refer to [15] for progressive censoring schemes and their related issues, see also [16,17]. Recently several authors were interested in studying parameter inference for different lifetime distributions under progressive Type-II censoring scheme, see, for example, [18–23] in this respect.

Progressive Type-II censoring scheme can be described as follows: Let X1, X2, …, Xn denote the numerical outcomes of n independent and identically distributed (i.i.d) units from a life-test experiment. Suppose that R1, R2, …, Rm (m < n) are some fixed nonnegative integers such that $\sum_{i=1}^{m}R_{i}=n-m$, hence only m units will be observed, the remaining n-m units will be censored progressively according to the censoring scheme R = (R1, R2, …, Rm). The censoring occurs progressively in m stages, and the failure times of the m observed units are obtained at these stages. When the first failure (the first stage) *X*_1:*m*:*n*_ occurs, R1 of the n-1 surviving units are randomly censored from the experiment. When the second failure (the second stage) *X*_2:*m*:*n*_ occurs, R2 of the n-2-R1 surviving units are randomly censored from the experiment. Finally, at the time of the *m*^*th*^ failure (the *m*^*th*^ stage) *X*_*m*:*m*:*n*_, all the remaining Rm = n-m- (R1 + R2 + …+Rm-1) units are withdrawn from the experiment. It is obvious that Type-II right censoring and complete sampling schemes are special cases of progressive Type-II censoring scheme by choosing (R1 = R2 = … = Rm 1 = 0; Rm = n-m) and (R1 = R2 = … = Rm-1 = 0, n = m) respectively.

From our literature survey we realized that most of the work on the Marshall-Olkin Pareto distribution have been based on complete samples, also the idea of predicting censored units has not received much attention. This motivates us to write this article with two main objectives. The first objective is to provide the statistical inference about the unknown parameters of the Marshall-Olkin Pareto distribution when the lifetime data are observed under Progressive type-II censoring. The second objective is to provide the statistical inference about the censored observations. We consider both problems of estimation and prediction under classical as well as Bayesian approaches. Further, predictive interval estimates are also constructed.

The probability density function (pdf) and the cumulative distribution function (cdf) of MOP distribution are given respectively as:

$$f(x;\alpha ,\theta ,\beta )=\frac{\alpha \theta \beta^{\theta }x^{-(\theta +1)}}{{\left(1-\bar{\alpha }{\left(\frac{\bar{\beta }}{x}\right)}^{\theta }\right)}^{2}};\;\alpha ,\theta ,\beta >0,\;x\geq \beta .$$ (1)

$$F(x;\alpha ,\theta ,\beta )=1-\frac{a{\left(\frac{\beta }{x}\right)}^{\theta }}{1-\bar{\alpha }{\left(\frac{\beta }{x}\right)}^{\theta }};\;x\geq \beta$$ (2)

Prediction is very important in statistical inference, some prediction problems were discussed in the literature, see for example [24–30]. The main idea is predicting the future value of the ordered statistics based on the observed sample. [24] Used Bayes prediction to predict future values of a progressive censored sample under flexible Weibull distribution. [25] compare two prediction methods to predict the unobserved units from new Pareto model with progressive Type-II censoring scheme. In [30] the prediction of the remaining time for the generalized Pareto distribution under a progressive censored sample was considered.

The methodology in this paper is divided into two main objectives: First, find the Maximum likelihood estimator for the MOP parameters using the EM Algorithm, and use Bayes method to estimate of MOP parameters. The sample under consideration is a progressive Type-II censored sampling scheme. For Bayes estimation Gamma priors and quadratic loss function are considered. We compare the performance of the two methods of estimation numerically by simulation analysis using the R code. Second, we consider the prediction problem for the future unobserved data based on the available observation. Therefore, two prediction methods are performed (i) The best unbiased predictor (BUP) and (ii) The Bayes prediction (BP), also predictive intervals (PIs) of the future censored data are constructed. Numerical analysis and simulation are used for comparing the efficiency of the two prediction methods under consideration, and finally an illustrative real data of failure time example is presented.

The rest of this paper is organized as follows: In Section 2, the estimation methods including the MLE and the Bayesian estimation methods for estimating the three parameters of MOP distribution are computed using the EM algorithm and MCMC numerical methods. Also, we discuss the prediction methods for the non-observations from the censored sample using PUB and BP. In Section 3 the real data set is given for illustrative purposes, and numerical simulation study and its results are performed and summarized by tables and figures. Discussions are reported in Section 4, while conclusions are given in Section 5.

## 2. Materials and methods

### 2.1. Maximum likelihood inference

A well-known classical method of point estimation is used, which is the maximum likelihood method (MLE) for estimating the three unknown parameters of MOP distribution under progressive type-II censoring scheme. Let X = (*x*_1:*m*:*n*_, *x*_2:*m*:*n*_,…, *x*_m:*m*:*n*_) with *x*_1:*m*:*n*_≤ *x*_2:*m*:*n*_≤… ≤*x*_m:*m*:*n*_ be the observed progressive Type-II censored sample of size m drawn from a sample of size n under MOP distribution with the pdf and the cdf given by Eq (1) and Eq (2) respectively. The likelihood function under a progressive Type-II censored sample X is given by:

$$RL(\alpha ,\beta ,\theta ;X)=A\overset{m}{\underset{i=1}{\prod }}f(x_{i:m:n}){\left[1-F(x_{i:m:n})\right]}^{R_{i}}$$ (3)

where A = n(n-1-R1) (n-2-R1-R2) … (n-m+1-R1-…- Rm-1). See [15].

Using Eq (1) and Eq (2) we obtain:

$$L(\alpha ,\beta ,\theta ;X)=A\alpha^{n}\beta^{\theta n}\theta^{m}\overset{m}{\underset{i=1}{\prod }}\frac{x_{i:m:n}^{-\theta (1+R_{i})-1}}{{\left(1-\bar{\alpha }{\left(\frac{\beta }{x_{i:m:n}}\right)}^{\theta }\right)}^{2+R_{i}}}$$ (4)

The log-likelihood function of MOP is:

$$\begin{array}{c} l(\alpha ,\beta ,\theta ;X)=\log A+\mathrm{nlog}\alpha +\theta \mathrm{nlog}\beta +\mathrm{mlog}\theta -\sum_{i=1}^{m}(\theta (1+R_{i})+1)\log x_{i:m:n}-\\ \sum_{i=1}^{m}(2+R_{i})\log\left(1-\bar{\alpha }{\left(\frac{\beta }{x_{i:m:n}}\right)}^{\theta }\right) \end{array}$$

From the log-likelihood equation we can compute the derivatives with respect to the parameters, since *x*≥*β*, then the MLE of the parameter *β* is *x*_1:*m*:*n*_, where *x*_1:*m*:*n*_, is the first progressive censored statistic. We need to solve the following normal equations after equating them to zero:

$$\begin{array}{l} \frac{\partial l}{\partial \alpha }=\frac{n}{\alpha }-\sum_{i=2}^{m}(2+R_{i})\frac{{\left(\frac{x_{1:m:n}}{x_{i:m:n}}\right)}^{\theta }}{\left(1-\bar{\alpha }{\left(\frac{x_{1:m:n}}{x_{i:m:n}}\right)}^{\theta }\right)}=0\\ \frac{\partial l}{\partial \theta }=\frac{m}{\theta }+n\;\log\;x_{1:m:n}-\sum_{i=2}^{m}(1+R_{i})\log\;x_{i:m:n}+\sum_{i=2}^{m}(2+R_{i})\frac{\log(\frac{x_{1:m:n}}{x_{i:m:n}})}{\left(\frac{1}{\bar{\alpha }}{\left(\frac{x_{1:m:n}}{x_{i:m:n}}\right)}^{-\theta }-1\right)}=0 \end{array}$$

Since the closed-form solution for the above equations cannot be obtained explicitly, one needs to employ some numerical method. The most commonly used method in the literature is Newton-Raphson (N-R). But the main drawback of this method is that it requires the second derivatives of the log-likelihood function at all iterations, and it may be computationally cumbersome due to the complicated form of the likelihood function. Instead, numerical packages in various programming languages can also be used to solve the above equations. One of such good algorithms is the Expectation-maximization (EM) algorithm which had been used by several authors to obtain maximum likelihood estimates. This algorithm is very much useful compared to N-R method especially when data are not completely observed under some censoring scheme.

The normal approximation of the MLE can be used to construct approximate confidence intervals on the parameters *α*, *β* and *θ*. From the asymptotic property of the MLE we have $\sqrt{n}(\hat{\phi }-\phi {)}_{\to }^{D}N_{3}(0,I^{-1}(\phi ))$, where *ϕ* = (*α*, *β*, *θ*) and *I*(Φ) is the Fisher information matrix given by:

$$I(\Phi )=-\frac{1}{n}[\begin{array}{c} E(l_{\alpha \alpha })\;E(l_{\alpha \beta })\;E(l_{\alpha \theta })\\ E(l_{\beta \alpha })\;E(l_{\beta \beta })\;E(l_{\beta \theta })\\ E(l_{\theta \alpha })\;E(l_{\theta \beta })\;E(l_{\theta \theta }) \end{array}]$$

where the second partial derivatives are given as follow:

$$\begin{array}{l} l_{\alpha \alpha }=-\frac{n}{\alpha^{2}}+\sum_{i=2}^{m}(2+R_{i})\frac{\left(\frac{x_{1:m:n}}{{x_{i:m:n})}^{2\theta }}\right)}{{\left(1-\bar{\alpha }{\left(\frac{x_{1:m:n}}{x_{i:m:n}}\right)}^{\theta }\right)}^{2}}\\ l_{\alpha \beta }=0\\ l_{\alpha \theta }=-\sum_{i=2}^{m}(2+R_{i})\frac{\log(\frac{x_{1:m:n}}{x_{i:m:n}}){\left(\frac{x_{1:m:n}}{x_{i:m:n}}\right)}^{\theta }}{\left(1-\bar{\alpha }{\left(\frac{x_{1:m:n}}{x_{i:m:n}}\right)}^{\theta }\right)}\\ l_{\beta \beta }=-\frac{\theta n}{\beta^{2}}+\sum_{i=1}^{m}(2+R_{i})\frac{(1-\bar{\alpha }{\left(\frac{\beta }{x_{i:m:n}}\right)}^{\theta })\frac{\bar{\alpha }\theta (\theta -1)}{\beta^{2}}{\left(\frac{\beta }{x_{i:m:n}}\right)}^{\theta }+{\left(\frac{\bar{\alpha }\theta }{\beta }{\left(\frac{\beta }{x_{i:m:n}}\right)}^{\theta }\right)}^{2}}{{\left(1-\bar{\alpha }{\left(\frac{\beta }{x_{i:m:n}}\right)}^{\theta }\right)}^{2}}\\ l_{\beta \theta }=\frac{n}{\beta }+\sum_{i=1}^{m}(2+R_{i})\frac{\frac{\bar{\alpha }}{\beta }(1-\bar{\alpha }{\left(\frac{\beta }{x_{i:m:n}}\right)}^{\theta }){\left(\frac{\beta }{x_{i:m:n}}\right)}^{\theta }[\theta \log(\frac{\beta }{x_{i:m:n}})+1]+\frac{{\bar{\alpha }}^{2}\theta }{\beta }{\left(\frac{\beta }{x_{i:m:n}}\right)}^{2\theta }\log(\frac{\beta }{x_{i:m:n}})}{{\left(1-\bar{\alpha }{\left(\frac{\beta }{x_{i:m:n}}\right)}^{\theta }\right)}^{2}}\\ l_{\theta \theta }=-\frac{m}{\theta^{2}}+\sum_{i=2}^{m}(2+R_{i})\frac{\log(\frac{x_{1:m:n}}{x_{i:m:n}}){\left(\frac{x_{1:m:n}}{x_{i:m:n}}\right)}^{-\theta }\log(\frac{x_{1:m:n}}{x_{i:m:n}})}{\left({\left(\frac{x_{1:m:n}}{x_{i:m:n}}\right)}^{-\theta }-\bar{\alpha }\right)} \end{array}$$

The expected values of the second partial derivatives are obtained numerically using R-programming. The variances of the MLEs can be found from the asymptotic property of MLE so that $V({\hat{\alpha }}_{MLE})\approx \frac{E(l_{\beta \beta })E(l_{\theta \theta })-E^{2}(l_{\beta \theta })}{\mathrm{Det}\left(I(\Phi )\right)}$, $V({\hat{\beta }}_{MLE})\approx \frac{E(l_{\alpha \alpha })E(l_{\theta \theta })-E^{2}(l_{\alpha \theta })}{\mathrm{Det}\left(I(\Phi )\right)}$ and $V({\hat{\theta }}_{MLE})\approx \frac{E(l_{\alpha \alpha })E(l_{\beta \beta })-E^{2}(l_{\alpha \beta })}{\mathrm{Det}\left(I(\Phi )\right)}$, where Det(*I*(Φ)) is the determinant of the information matrix *I*. The (1−*ζ*)100% approximate confidence intervals for ${\hat{\alpha }}_{MLE},\;{\hat{\beta }}_{MLE}$, and ${\hat{\theta }}_{MLE}$ are given respectively as:

$${\hat{\alpha }}_{MLE}\pm z_{\frac{\zeta }{2}}\sqrt{V({\hat{\alpha }}_{MLE})},\;{\hat{\beta }}_{MLE}\pm z_{\frac{\zeta }{2}}\sqrt{V({\hat{\beta }}_{MLE})},\;{\hat{\theta }}_{MLE}\pm z_{\frac{\zeta }{2}}\sqrt{V({\hat{\theta }}_{MLE})}$$

### 2.2. EM algorithm

The basic idea of EM algorithm begins with writing the log-likelihood function given the complete sample W. However, we have the observed data X = (*x*_1:*m*:*n*_, *x*_2:*m*:*n*_,…, *x*_*m*:*m*:*n*_), while *n*−*m* units will be removed or censored. Now, suppose that the lifetimes of the censored observations are Z = (*z*_1_, *z*_2_,…,*z*_*n*−*m*_), hence the complete sample W is a combination of the observed data X and censored data Z; that means W = (X, Z). Dealing with the complete log-likelihood function and differentiate it partially with respect to the parameters *α* and *θ* and equate them to zero, we get:

$$\begin{array}{l} \frac{\partial l}{\partial \alpha }=\frac{n}{\alpha }-\sum_{i=2}^{m}(2+R_{i})\frac{{\left(\frac{x_{1:m:n}}{x_{i:m:n}}\right)}^{\theta }}{\left(1-\bar{\alpha }{\left(\frac{x_{1:m:n}}{x_{i:m:n}}\right)}^{\theta }\right)}+2\sum_{i=1}^{n-m}\frac{{\left(\frac{x_{1:m:n}}{z_{i}}\right)}^{\theta }}{\left(1-\bar{\alpha }{\left(\frac{x_{1:m:n}}{z_{i}}\right)}^{\theta }\right)}\\ \frac{\partial l}{\partial \theta }=\frac{n}{\theta }+nlog\;x_{1:m:n}-\sum_{i=2}^{m}(1+R_{i})log\;x_{i:m:n}+\sum_{i=2}^{m}(2+R_{i})\frac{\log(\frac{x_{1:m:n}}{x_{i:m:n}})}{\left(\frac{1}{\bar{\alpha }}(\frac{x_{1:m:n}}{{\left(x_{i:m:n}\right)}^{-\theta }-1)})\right)}-\sum_{i=1}^{n-m}log\;(z_{i})+2\sum_{i=1}^{n-m}\frac{\log\;(\frac{x_{1:m:n}}{z_{i}})}{\left(\frac{1}{\bar{\alpha }}{\left(\frac{x_{1}\cdot m_{i}\cdot n}{z_{i}}\right)}^{-\theta }-1\right)} \end{array}$$ (5)

When applying the EM algorithm, we should keep in mind that the MLE of *β* is *x*_1:*m*:*n*_ so it will be considered as a known parameter in the above normal equations. The EM algorithm consists of an expected step (E-step) and a maximized step (M-step). The E-step replaces the expressions of observed and censored lifetimes by their expectations, whereas M-step maximizes the E-step at each iteration.

### 2.3. Bayes estimation

In the Bayesian method, all parameters are considered as random variables with a certain distribution called prior distribution. If prior information is not available which is usually the case, we need to select one. Since selecting prior distribution has an important role in the estimation of the parameters, our selection for the priors of α, *β*, *and θ* are the independent gamma distributions G(a_1_; b_1_); G(a_2_; b_2_) and G(a_3_; b_3_) respectively. The reason for choosing this prior density is that Gamma prior has flexible nature as a non-informative prior, especially when the values of the hyperparameters are assumed to be zero. The suggested gamma distributions have the following densities:

$$\begin{array}{c} g_{\alpha }(a_{1},b_{1})=\frac{b_{1}^{a_{1}}}{\Gamma (a_{1})}\alpha^{a_{1}-1}e^{-b_{1}\alpha }\\ g_{\beta }(a_{2},b_{2})=\frac{b_{2}^{a_{2}}}{\Gamma (a_{2})}\beta^{a_{2}-1}e^{-b_{2}\beta }\\ g_{\theta }(a_{3},b_{3})=\frac{b_{3}^{a_{3}}}{\Gamma (a_{3})}\theta^{a_{3}-1}e^{-b_{3}\theta } \end{array}$$ (6)

where *a*_1_, *a*_2_, *a*_3_, *b*_1_, *b*_2_ and *b*_3_ are hyperparameters of prior distributions and all are positive real constants. The joint prior of α, *β*, *and θ* is:

$$g(\alpha ,\beta ,\theta )\propto \alpha^{a_{1}-1}\beta^{a_{2}-1}\theta^{a_{3}-1}e^{-b_{1}\alpha -b_{2}\beta -b_{3}\theta },\alpha ,\beta ,\theta ,a_{1},a_{2},a_{3},b_{1},b_{2},b_{3}>0$$

and the joint posterior of α, *β*, *and θ* is:

$$p(\alpha ,\beta ,\theta |x)=\frac{L(\frac{\underline{x}}{\alpha },\theta ,\beta )g(\alpha ,\theta ,\beta )}{\int_{\alpha }\int_{\theta }\int_{\beta }L(\frac{\underline{x}}{\alpha },\theta ,\beta )g(\alpha ,\theta ,\beta )d\alpha d\theta d\beta }$$ (7)

where *L*(*x*/*α*, *θ*, *β*) is the likelihood function of MOP distribution under progressive type-II censored samples as in Eq (4) Substituting *L*(*x*/*α*, *θ*, *β*) and *g*(*α*, *β*, *θ*) for MOP under progressive Type-II censoring scheme, the joint posterior density can be written as:

$$\begin{array}{c} p(\alpha ,\beta ,\theta |x)\propto \alpha^{a_{1}-1}\beta^{a_{2}-1}\theta^{a_{3}-1}e^{-b_{1}\alpha -b_{2}\beta -b_{3}\theta }\theta^{m}\alpha^{n}\beta^{\theta n}\times \\ \prod_{i=1}^{m}\frac{x_{i:m:n}^{-\theta (1+R_{i})-1}}{{\left(1-\bar{\alpha }{\left(\frac{\beta }{x_{i:m:n}}\right)}^{\theta }\right)}^{2+R_{i}}}\\ \propto G_{\alpha }(a_{1}+n,b_{1})G_{\beta /\theta }(a_{2}+\theta n,b_{2})G_{\theta }(a_{3}+m,b_{3})Q(\alpha ,\beta ,\theta ) \end{array}$$ (8)

where $Q(\alpha ,\beta ,\theta )=\prod_{i=1}^{m}\frac{x_{i:m:n}^{-\theta (1+R_{i})-1}}{{\left(1-\bar{\alpha }{\left(\frac{\beta }{x_{i:m:n}}\right)}^{\theta }\right)}^{2+R_{i}}}$, and G(.,.) represents the probability density of Gamma distribution.

Therefore, the Bayes estimate of any function of α, *β*, *and θ*, say *h*(*α*, *β*, *θ*), under the quadratic loss function will be the expected value of *h*(*α*, *β*, *θ*), i.e. h^(α,β,θ)=Eα,β,θdata(h(α,β,θ)). Since it is difficult to compute this expected value analytically, we opt to use the Markov chain Monte Carlo technique (MCMC), see [31] and [32].

The Gibbs sampling method will be used to generate a sample from the posterior density function *p*(*α*, *β*, *θ*|x) and compute Bayes estimates. To generate a sample from the posterior distribution, it is assumed that the pdf of prior density is as described in Eq (6). The fully conditional posterior densities of α, *β*, *and θ*, and the data is given by:

$$\begin{array}{l} \pi (\alpha |\beta ,\theta ,x)\;\propto G_{\alpha }(a_{1}+n,b_{1})\prod_{i=1}^{m}\frac{1}{{\left(1-\bar{\alpha }{\left(\frac{\beta }{x_{i:m:n}}\right)}^{\theta }\right)}^{2+R_{i}}},\\ \pi (\beta |\alpha ,\theta ,x)\;\propto G_{\beta /\theta }(a_{3}+\theta n,b_{3})\prod_{i=1}^{m}\frac{1}{{\left(1-\bar{\alpha }{\left(\frac{\beta }{x_{i:m:n}}\right)}^{\theta }\right)}^{2+R_{i}}}\\ \pi (\theta |\alpha ,\beta ,x)\;\propto G_{\theta }(a_{2}+m,b_{2})Q(\alpha ,\beta ,\theta ). \end{array}$$ (9)

Since these full conditional distributions cannot be reduced to well-known distributions, we cannot generate α, *β*, *and θ* from these distributions directly by standard methods, therefore we need to generate these distributions using the M-H algorithm, see [33] and [34]. The idea here is to decrease the rate of rejections as much as possible. The algorithm below depends on using the M-H algorithm based on choosing the normal distribution as a proposal distribution which is used to find the Bayes estimators and also to construct the credible intervals for α, *β*, *and θ*. To apply the Gibbs technique, we need the following algorithm:

1. Start with initial values (*α*^0^, *β*^0^, *θ*^0^)
2. Use the M-H algorithm to generate a posterior sample for α, *β*, *and θ* from Eq (8).
3. Repeat step 2 M times and obtain (*α*_1_, *β*_1_, *θ*_1_); (*α*_2_, *β*_2_, *θ*_2_), …, (*α*_*M*_, *β*_*M*_, *θ*_*M*_).
4. After obtaining the posterior sample, the Bayes estimates of α, *β*, *and θ* concerning quadratic loss function are:

$$\begin{array}{c} {\hat{\alpha }}^{MC}=[E_{\pi }(\alpha |x)]\approx (\frac{1}{M-M_{0}}\sum_{i=1}^{M-M_{0}}\alpha_{i})\\ {\hat{\beta }}^{MC}=[E_{\pi }(\beta |x)]\approx (\frac{1}{M-M_{0}}\sum_{i=1}^{M-M_{0}}\beta_{i})\\ {\hat{\theta }}^{MC}=[E_{\pi }|\theta x)]\approx (\frac{1}{M-M_{0}}\sum_{i=1}^{M-M_{0}}\theta_{i}) \end{array}$$ (10)

where, M_0_ is the burn-in-period of Markov Chain.

### 2.4 Prediction

In many fields of life testing and reliability studies predicting the unobserved or censored observation from the observed sample data has a great attention, see for example [24–30]. In this section, we perform two prediction methods, namely the best-unbiased predictor (BUP) and the Bayes predictor (BP).

#### 2.4.1. Best unbiased predictor

In this section our goal is to predict the lifetimes of the *s*^*th*^ order $Y_{s:r_{j}}$ (s = 1, 2, …, r_j_; j = 1, 2, …, m) from the observations under progressive type-II censored sample, X = (*x*_1:*m*:*n*_, *x*_2:*m*:*n*_,…, *x*_*m*:*m*:*n*_). Now by using the Markovian property of Progressive type-II censored order statistics, $Y_{s:r_{j}}/X=x$ acts similar to the *s*^*th*^ order statistic drawn from a sample of size *r*_*j*_ under truncated distribution at *x*_*j*:*m*:*n*_ with pdf $\frac{f(y)}{\left(1-F(x_{j:m:n}\right)},$ *y*>*x*_*j*:*m*:*n*_, hence we obtain:

$$\begin{array}{c} f_{Y_{s:r_{j}}|X}(y_{s:r_{j}};\alpha ,\beta ,\theta )=f_{Y_{s:r_{j}}|x_{j:m:n}}(y_{s:r_{j}};\alpha ,\beta ,\theta )\\ =c^{*}{\left[F(y_{s:r_{j}})-F(x_{j:m:n})\right]}^{s-1}{\left[1-F(y_{s:r_{j}})\right]}^{r_{j}-s}\frac{f(y_{s:r_{j}})}{{\left[1-F(x_{j:m:n})\right]}^{r_{j}}},y_{s:r_{j}}>x_{j:m:n} \end{array}$$ (11)

where $c^{*}=\frac{r_{j}!}{(s-1)!(r_{j}-s)!}$. Now substituting the pdf and the cdf of MOP distribution into Eq (11) and after some simplifications we observe:

$$f_{Y_{s:r_{j}}|X}(y_{s:r_{j}};\alpha ,\beta ,\theta )=c^{*}\theta \frac{{\left(x_{j:m:n}^{-\theta }-y_{s:r_{j}}^{-\theta }\right)}^{s-1}y_{s:r_{j}}^{-\theta (r_{j}-s+1)-1}x_{j:m:n}^{\theta r_{j}}}{{\left(1-\bar{\alpha }{\left(\frac{\beta }{x_{j:m:n}}\right)}^{\theta }\right)}^{s-r_{j}-1}{\left(1-\bar{\alpha }{\left(\frac{\beta }{y_{s:r_{j}}}\right)}^{\theta }\right)}^{r_{j}+1}}$$ (12)

The conditional density in Eq (12) can be rewritten using the well-known binomial expansion to become:

$$f_{Y_{s:r_{j}}|X}(y_{s:r_{j}}|\alpha ,\beta ,\theta )=c^{*}\theta \frac{\sum_{k=0}^{s-1}(-1{)}^{k}(\begin{array}{c} s-1\\ k \end{array})y_{s:r_{j}}^{-\theta (k+r_{j}-s+1)-1}x_{j:m:n}^{\theta (r_{j}-s+k+1)}}{{\left(1-\bar{\alpha }{\left(\frac{\beta }{x_{j:m:n}}\right)}^{\theta }\right)}^{s-r_{j}-1}{\left(1-\bar{\alpha }{\left(\frac{\beta }{y_{s:r_{j}}}\right)}^{\theta }\right)}^{r_{j}+1}}$$ (13)

The best unbiased predictor (BUP) of $Y_{s:r_{j}}$ is the expected value $E(Y_{s:r_{j}}/X_{j:m:n})$ that is:

$$\begin{array}{l} E(Y_{s:r_{j}}|X_{j:m:n})=|\int_{x_{j:m:n}}^{\infty }y_{s:r_{j}}f_{Y_{o:r_{j}}|X}(Y_{s:r_{j}};\alpha ,\beta ,\theta )dy_{s:r_{j}}\\ \;=\frac{c^{*}\theta \sum_{s-1}^{k=0}{\left(-1\right)}^{k(\begin{array}{c} s-1\\ k \end{array})x_{j:m:n}^{\theta (r_{j}-s+k+1)}}}{{\left(1-\bar{\alpha }{\left(\frac{\beta }{x_{j:m:n}}\right)}^{\theta }\right)}^{s-r_{j}-1}}\int_{x_{j:m:n}}^{\infty }\frac{y_{s:r_{j}}^{-\theta (k+r_{j}-s+1)}}{{\left(1-\bar{\alpha }{\left(\frac{\beta }{y_{s:r_{j}}}\right)}^{\theta }\right)}^{r_{j}+1}}dy_{s:r_{j}} \end{array}$$ (14)

Using integration techniques and binomial expansion, Eq (14) will reduce to censored data. In Bayes estimation, we assume:

$$E(Y_{s:r_{j}}|X_{j:m:n})=\{\begin{array}{c} c^{*}\sum_{i,k}\frac{(u-\begin{array}{c} \frac{1}{\theta }\\ i \end{array}-1){\left(x_{j:m:n}\right)}^{\theta u}{\left(1-\Delta \right)}^{i-r_{j}}}{{\bar{\alpha }}^{u-\frac{1}{\theta }}\beta^{\theta u-1}(i-r_{j}){\left(\Delta \right)}^{k-u}},0<\alpha <1\\ c^{*}\sum_{i,k}\frac{(u-\begin{array}{c} \frac{1}{\theta }\\ i \end{array}-1){\left(x_{j:m:n}\right)}^{\theta u}{\left(1-\Delta \right)}^{k-i-s-\frac{1}{\theta }})}{{\bar{\alpha }}^{u-\frac{1}{\theta }}\beta^{\theta u-1}(k-i-s-\frac{1}{\theta }){\left(\Delta \right)}^{k-u}},\alpha >1 \end{array}$$

where $\sum_{i,k}=\sum_{i=0}^{\infty }\sum_{k=0}^{\infty }\left(-1{)}^{k+i}(\begin{array}{c} s-1\\ k \end{array}\right),\;\Delta =1-\bar{\alpha }{\left(\frac{\beta }{x_{j:m:n}}\right)}^{\theta }$ and *u* = *k*+*r*_*j*_−*s*+1. If it is assumed that the parameters α, *β*, *and θ* are all unknown, then the BUP of $Y_{s:r_{j}}$ will be:

$${\hat{Y}}_{s:r_{j}}=\{\begin{array}{c} c^{*}\sum_{i,k}\frac{(u-\begin{array}{c} \frac{1}{\hat{\theta }}\\ i \end{array}-1){\left(x_{j:m:n}\right)}^{\hat{\theta }u}{\left(1-\hat{\Delta }\right)}^{i-r_{j}}}{{\hat{\bar{\alpha }}}^{u-\frac{1}{\hat{\theta }}\hat{\beta }\hat{\theta }u-1}(i-r_{j}){\left(\hat{\Delta }\right)}^{k-u}},0<\alpha <1\\ c^{*}\sum_{i,k}\frac{(u-\begin{array}{c} \frac{1}{\hat{\theta }}\\ i \end{array}-1){\left(x_{j:m:n}\right)}^{\hat{\theta }u}{\left(1-\hat{\Delta }\right)}^{k-i-s-\frac{1}{\hat{\theta }}})}{{\hat{\bar{\alpha }}}^{u-\frac{1}{\hat{\theta }}\hat{\beta }}\hat{\theta }u-1(k-i-s-\frac{1}{\hat{\theta }}){\left(\hat{\Delta }\right)}^{k-u}},\alpha >1 \end{array}$$

where $\hat{\alpha },\;\hat{\beta },$ and $\hat{\theta }$ are the MLEs of α, *β*, *and θ* respectively and $\hat{\Delta }=1-\hat{\bar{\alpha }}{\left(\frac{\hat{\beta }}{x_{j:m:n}}\right)}^{\hat{\theta }}$.

#### 2.4.2 Bayesian prediction

The Bayes prediction of the non-observed units from the future sample depends on the current observed sample which is called an informative sample. For that purpose, we obtain the estimation of the posterior predictive density of the *s*^*th*^ order $Y_{s:r_{j}}$. The posterior predictive density of $Y_{s:r_{j}}$ given the observed censored data X is given by:

$$\pi (Y_{s:r_{j}}|X)=\int_{0}^{\infty }\int_{0}^{\infty }\int_{0}^{\infty }f_{Y_{s:r_{j}}|X}(y_{s:r_{j}}|\alpha ,\beta ,\theta )p(\alpha ,\beta ,\theta |x)d\alpha d\beta d\theta ,y_{s:r_{j}}>x_{j:m:n}$$ (15)

where $f_{Y_{s:r_{j}}|X}(y_{s:r_{j}}|\alpha ,\beta ,\theta )$ is the conditional density of $Y_{s:r_{j}}$ given *α*, *β*, *θ*, and the data X, which is given in Eq (13), where *p*(*α*, *β*, *θ*|x) is the joint posterior given in Eq (8). Using the squared error loss function (SEL), the Bayes predictor (BP) of $Y=Y_{s:r_{j}}$ is obtained by:

$$\begin{array}{l} Y_{s:r_{j}}^{BP}=E_{\pi }(Y|\mathrm{data})\\ \;=c^{*}\int_{x_{j:m:n}}^{\infty }y\int_{0}^{\infty }\int_{0}^{\infty }\int_{0}^{\infty }\frac{\sum_{s-1}^{k=0}{\left(-1\right)}^{k}(\begin{array}{c} s-1\\ k \end{array})y^{-\theta u-1}x_{j:m:n}^{\theta u}}{\Delta^{s-r_{j}-1}{\left(1-\bar{\alpha }{\left(\frac{\beta }{y}\right)}^{\theta }\right)}^{r_{j}+1}}\alpha^{n+a_{1}-1}\theta^{a_{2}+m}\beta^{\theta n+a_{3}-1}e^{-b_{1}\alpha -b_{2}\theta -b_{3}\beta }\times \\ \;\prod_{i=1}^{m}\frac{x_{j:m:n}^{-\theta (1+R_{i})-1}}{\Delta^{2+R_{i}}}d\alpha d\beta d\theta dy \end{array}$$ (16)

The form of the posterior predictive density in Eq (16) is not easy to compute, therefore evaluating the predictive Bayes estimates *E*_*π*_(*Y*|data) manually is not an easy task, hence we use numerical techniques such as MCMC samples that was described in Section. 3 to generate samples from the predictive distributions and find the Bayes predictor.

Based on MCMC samples {(*α*_ℓ_, *β*_ℓ_, *θ*_ℓ_): ℓ = 1,2,…*M*} that are obtained by using the Gibbs sampling and M-H methods, the Bayes predictor ${\hat{Y}}_{s:r_{j}}^{BP}$ is now given by:

$$\begin{array}{l} {\hat{Y}}_{S:r_{j}}^{BP}=\frac{c^{*}}{M}\sum_{l=1}^{M}\frac{\sum_{s-1}^{k=0}{\left(-1\right)}^{k}(\begin{array}{c} s-1\\ k \end{array})x_{j:m:n}^{\theta_{l}u}}{\Lambda^{k-u}}\alpha_{l}^{n+a_{1}-1}\theta_{l}^{a_{2}+m}\beta_{l}^{\theta_{l}n+a_{3}-1}e^{-b_{1}\alpha_{l}-b_{2}\theta_{l}-b_{3}\beta_{l}}\times \\ \;\prod_{i=1}^{m}\frac{x_{j:m:n}^{-\theta_{l}(1+R_{i})-1}}{\Lambda^{2+R_{i}}}\int_{x_{j:m:n}}^{\infty }y^{-u\theta_{l}}{\left(1-{\bar{\alpha }}_{l}{\left(\frac{\beta_{l}}{y}\right)}^{\theta_{l}}\right)}^{-r_{j}-1}dy, \end{array}$$ (17)

where $\sum_{l,k,t}=\sum_{l=1}^{M}\sum_{k=0}^{s-1}\sum_{t=0}^{\infty }(-1{)}^{k+t}\left(\begin{array}{c} s-1\\ k \end{array}\right)\alpha_{l}^{n+a_{1}-1}\theta_{l}^{a_{2}+m}\beta_{l}^{\theta_{l}n+a_{3}-1}e^{-b_{1}\alpha_{l}-b_{2}\theta_{l}-b_{3}\beta_{l}}$ and $\Lambda =1-{\bar{\alpha }}_{l}{\left(\frac{\beta_{l}}{x_{j:m:n}}\right)}^{\theta_{l}}$.

From the above posterior predictive density one can obtain a two-sided predictive interval for $Y=Y_{s:r_{j}}$, (s = 1, 2, …, *r*_*j*_; j = 1, 2, …, m) For that purpose, we need to find the predictive survival function of $Y=Y_{s:r_{j}}$ at any point *y*>*x*_*j*:*m*:*n*_, which can be defined as:

$$\begin{array}{l} S_{Y|\mathrm{data}}(y|\alpha ,\beta ,\theta )=\int_{y}^{\infty }f_{Y|\mathrm{data}}(z|\alpha ,\beta ,\theta )dz\\ \;=\left\{ \begin{array}{l} c^{*}\sum_{i=0}^{\infty }\sum_{k=0}^{s-1}\frac{{\left(-1\right)}^{k+i}(\begin{array}{c} s-1\\ k \end{array})(\begin{array}{c} u-1\\ i \end{array}){\left(x_{j:m:n}\right)}^{\theta u}(1-{\left(1-\bar{\alpha }{\left(\frac{\beta }{y}\right)}^{\theta }\right)}^{i-r_{j}})}{{\bar{\alpha }}^{u}\beta^{\theta u}(i-r_{j})\Delta^{k-u}},\;0<\alpha <1\\ c^{*}\sum_{i=0}^{\infty }\sum_{k=0}^{s-1}\frac{{\left(-1\right)}^{k+i}(\begin{array}{c} e-1\\ k \end{array})(\begin{array}{c} u-1\\ i \end{array}){\left(x_{j:m:n}\right)}^{\theta u}(1-{\left(1-\bar{\alpha }{\left(\frac{\beta }{y}\right)}^{\theta }\right)}^{k-i-s-\frac{1}{\theta }})}{{\bar{\alpha }}^{u}\beta^{\theta u}(k-i-s-\frac{1}{\theta })\Delta^{k-u}},\;\alpha >1 \end{array}\right. \end{array}$$

Under the SEL function, the predictive survival function of $Y=Y_{s:r_{j}}$ is given by:

$$\begin{array}{l} S_{Y\setminus \mathrm{data}}^{P}(y|\alpha ,\beta ,\theta )\\ =\left\{ \begin{array}{c} \int_{0}^{\infty }\int_{0}^{\infty }\int_{0}^{\infty }c^{*}\sum_{i,k}\frac{\left(\begin{array}{c} u-1\\ i \end{array}){\left(x_{j:m:n}\right)}^{\theta u}(1-{\left(1-\bar{\alpha }{\left(\frac{\beta }{y}\right)}^{\theta }\right)}^{i-r_{j}}\right)}{{\bar{\alpha }}^{u}\beta^{\theta u}(i-r_{j})\Delta^{k-u}}p(\alpha ,\theta ,\beta |x)d\alpha d\beta d\theta ,0<\alpha <1\\ \int_{0}^{\infty }\int_{0}^{\infty }\int_{0}^{\infty }c^{*}\sum_{i,k}\frac{\left(\begin{array}{c} u-1\\ i \end{array}){\left(x_{j:m:n}\right)}^{\theta u}(1-{\left(1-\bar{\alpha }{\left(\frac{\beta }{y}\right)}^{\theta }\right)}^{k-i-s-\frac{1}{\theta }}\right)}{{\bar{\alpha }}^{u}\beta^{\theta u}(k-i-s-\frac{1}{\theta })\Delta^{k-u}}p(\alpha ,\theta ,\beta |x)d\alpha d\beta d\theta ,\alpha >1 \end{array}\right. \end{array}$$ (18)

The predictive survival function in Eq (17) cannot be easily evaluated analytically, hence numerical approximation technique is preferable in this case. The MCMC samples can be used to approximately evaluate Eq (17), so let {(*α*_ℓ_, *β*_ℓ_, *θ*_ℓ_): ℓ = 1,2,…*M*}, then the simulated estimator for the predictive survival function is written as:

$${\hat{S}}_{Y\setminus \mathrm{data}}^{P}(y|\alpha ,\beta ,\theta )=\{\begin{array}{c} \frac{c^{*}}{M}\sum_{l,i,k}\frac{\left(\begin{array}{c} u-1\\ i \end{array}){\left(x_{j:m:n}\right)}^{\theta_{l}u}(1-{\left(1-{\bar{\alpha }}_{l}{\left(\frac{\beta_{l}}{y}\right)}^{\theta_{l}}\right)}^{i-r_{j}}\right)}{{\bar{\alpha }}_{l}^{u}\beta_{l}^{\theta_{l}u}(i-r_{j})\Lambda^{k-u}},0<\alpha <1\\ \frac{c^{*}}{M}\sum_{l,i,k}\frac{\left(\begin{array}{c} u-1\\ i \end{array}){\left(x_{j:m:n}\right)}^{\theta_{l}u}(1-{\left(1-{\bar{\alpha }}_{l}{\left(\frac{\beta_{l}}{y}\right)}^{\theta_{l}}\right)}^{k-i-s-\frac{1}{\theta_{l}}}\right)}{{\bar{\alpha }}_{l}^{u}\beta_{l}^{\theta_{l}u}(k-i-s-\frac{1}{\theta_{l}})\Lambda^{k-u}},\alpha >1 \end{array}$$

where $\sum_{l,i,k}=\sum_{l=1}^{M}\sum_{i=0}^{\infty }\sum_{k=0}^{s-1}(-1{)}^{k+i}\left(\begin{array}{c} s-1\\ k \end{array}\right)$.

Now, the (1−*ξ*)100% predictive interval of $Y=Y_{s:r_{j}}$ can be evaluated by solving the above non-linear equations with the lower bound (L) and the upper bound (U) using a suitable numerical technique.

${\hat{S}}_{Y\setminus data}^{P}(L)=1-\frac{\xi }{2}$ and ${\hat{S}}_{Y\setminus data}^{P}(U)=\frac{\xi }{2}$

## 3. Results

### 3.1 Real data

We consider a progressively censored real data set from [35], it consists of the failure times of 20 mechanical components, see Table 1.

**Table 1:** Data set of failure times of 20 mechanical components.

0.067	0.068	0.076	0.081	0.084	0.085	0.085	0.086	0.089	0.098
0.098	0.114	0.114	0.115	0.121	0.125	0.131	0.149	0.160	0.485

We examine the behavior of the estimators and predictors based on censored sample data from Table 1 Now, from the given data set we suggest three different progressive Type-II censoring schemes, these censoring schemes are:

1. scheme 1: (0 *(0.9 *n-1), 0.1 * n)
2. scheme 2: (0.1 * n, 0 * (0.9 * n—1))
3. scheme 3: (1 * (0.1 * n), (0 * 0.8 * n))

Based on these censored samples we estimate the parameters of the MOP distribution using MLE and Bayesian approaches. Table 2 shows the MLE computed using Newton-Raphson method and Bayes estimates under SEL obtained using MCMC technique and it also contains the 95% asymptotic confidence and credible interval estimates.

**Table 2:** MLE and Bayes point and interval estimate for real data sample.

Scheme	MLE			95% Asymptotic CI			Bayes			95% Credible CI		
n = 20, m = 18	$\hat{\alpha }$	$\hat{\beta }$	$\hat{\theta }$	$\hat{\alpha }$	$\hat{\beta }$	$\hat{\theta }$	$\hat{\alpha }$	$\hat{\beta }$	$\hat{\theta }$	$\hat{\alpha }$	$\hat{\beta }$	$\hat{\theta }$
Scheme 1 (0*17,2)	0.3323	0.34159	0.4008	(0.0234, 0.8980)	(0.0491, 0.7323)	(0.0780, 0.7237)	0.3324	0.3401	0.4096	(0.0196, 0.5206)	(0.0218, 0.5232)	(0.0414, 0.6415)
Scheme 2 (2,0*17)	0.3873	0.4089	0.4601	(0.1023, 0.5755)	(0.2655, 0.6588)	(0.30798, 0.8207)	0.3908	0.4152	0.4617	(0.09215, 0.4783)	(0.1512, 0.5832)	(0.2723, 0.7957)
Scheme 3 (1,1,0*16)	0.5132	0.6239	0.6711	(0.5903, 0.7071)	(0.5379, 0.8723)	(0.5504, 0.9635)	0.6143	0.7359	0.8032	(0.3662, 0.8093)	(0.4832, 0.9570)	(0.5198, 0.9810)

To check the validity of Marshall-Olkin Pareto (MOP) distribution to fit this data set, we use the Kolmogorov–Smirnov (K–S) test is applied. Which is the distance between the fitted and empirical distribution functions. The K–S distance and its respective p-value are computed to be K − S = 0.1304 and p-value = 0.8441, respectively. Therefore, it is quite reasonable to indicate that the MOP distribution is fitting this data well.

Table 3 reports some true values of $Y_{s:r_{j}}$ which are compared to predictive observations obtained using censoring scheme 1, 2, and 3, under BUP and BP methods of prediction. It can be shown clearly that the predicted values of $Y_{s:r_{j}}$ under BP method are closer to the real values than the BUP method, and this is true for all censoring schemes under consideration. Hence, we recommend using BP method for predicting the unobserved units in this real data example.

**Table 3:** True and predicted observations under censoring scheme 1, 2, and 3.

Scheme	True value of $Y_{s:r_{j}}$	Predicted value of $Y_{s:r_{j}}$ under BUP	Predicted value of $Y_{s:r_{j}}$ under BP
1	0.160 0.485	0.165 0.492	0.159 0.486
2	0.068 0.076	0.073 0.081	0.071 0.075
3	0.068 0.081	0.075 0.090	0.069 0.082

### 3.2. Simulation study

In this subsection, first, we use simulation analysis to check the performance of the Bayes estimators compared with the classical estimators obtained via the MLE approach under the progressive Type-II MOP censored sample. Second, we find the best unbiased predictor and Bayes prediction for the non-observed data based on observed ones. The squared error loss function SEL is used for Bayesian estimation. For the comparison needs, we use the mean square errors (MSEs) for the different estimators based on 10000 replications using R package. The generator of MOP distribution is $Q(p)=\beta {\left(\frac{1-\bar{\alpha }p}{1-p}\right)}^{1/\theta }$, where p is the uniform distribution deviates on (0,1). We obtain the point predictors and the 95% prediction intervals for the non-observed order statistics $Y_{s:r_{j}}$; (s = 1, 2, …, *r*_*j*_; j = 1, 2, …, m).

The MLE and Bayes estimators for the MOP parameters under the three censoring schemes are given in Tab. 4 and 5, with initial values of the parameters (*α*, *β*, *θ*) as (2,0.5,1.5) and (2.5,0.5,1.5).

**Fig 1:**
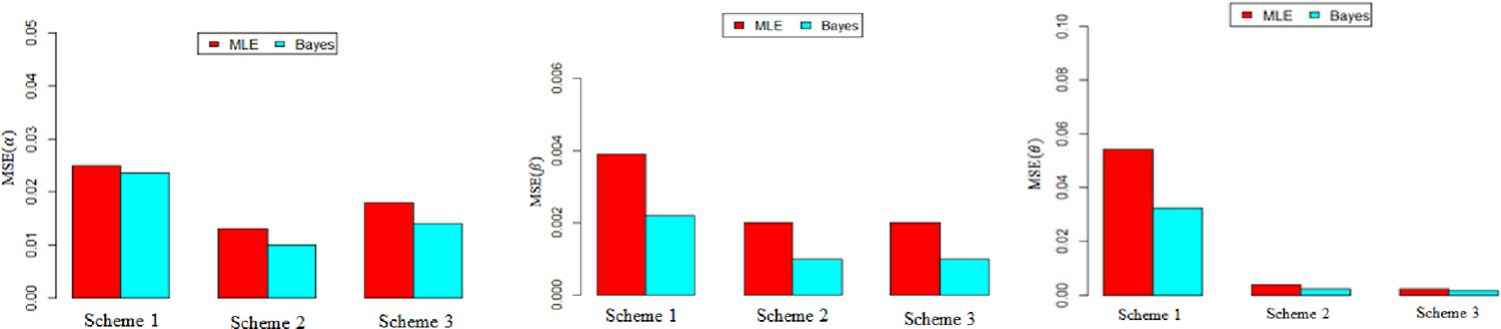
MSE of MOP parameters with various schemes with *n* = 50 and *α* = 2, *θ* = 1.5, *β* = 0.5.

**Fig 2:**
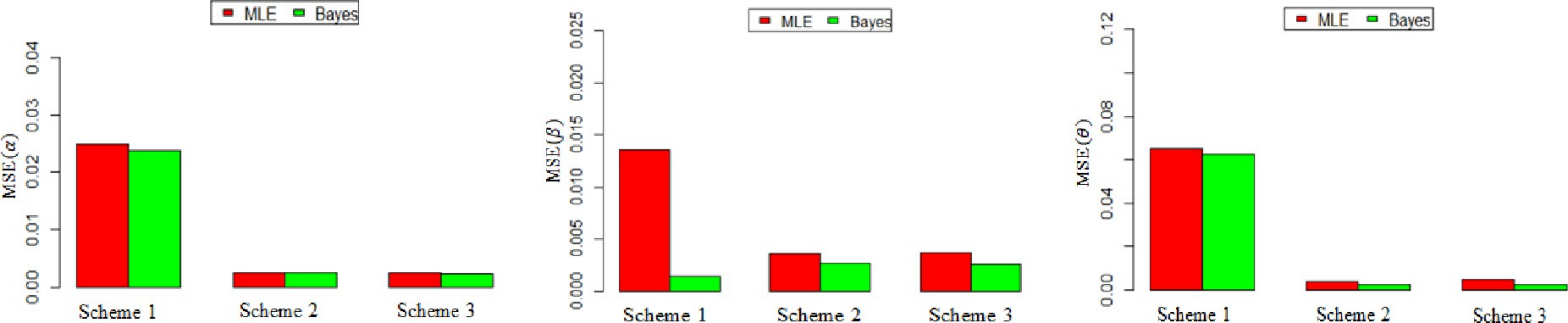
MSE of MOP parameters with various schemes with *n* = 50 and *α* = 2.5, *θ* = 1.5, *β* = 0.5.

The bias and the MSE are used to assess the performance of the two estimation methods. It can be viewed from Tables 4 and 5 and Figs (1) and (2) that the Bayesian estimation method performs better than the MLE with respect to Bias and MSE for estimating *α*, *β*, *and θ*, since the MSE and the bias have smaller values when using the Bayesian estimation method than their values under the MLE.

**Table 4:** MSEs and biases for MLEs and bayes estimators of *α* = 2, *θ* = 1.5, *β* = 0.5.

			MLE		Bayes (SEL)	
Scheme	n	Parameters	Bias	MSE	Bias	MSE
1	50	*α*	0.1116	0.0250	0.1107	0.0245
		*β*	0.0163	0.0039	0.0133	0.0022
		*θ*	0.1580	0.0543	0.1269	0.0322
	100	*α*	0.1120	0.0326	0.1277	0.0276
		*β*	0.0176	0.0052	0.0163	0.0031
		*θ*	0.1668	0.0715	0.1367	0.0427
2	50	*α*	0.0225	0.0013	0.0218	0.0012
		*β*	0.0035	0.0002	0.0007	0.0001
		*θ*	0.0290	0.0023	0.0217	0.0039
	100	*α*	0.0311	0.0018	0.0310	0.0012
		*β*	0.0042	0.0003	0.0035	0.0002
		*θ*	0.0356	0.0027	0.0307	0.0016
3	50	*α*	0.0224	0.0013	0.0217	0.0012
		*β*	0.0034	0.0002	0.0011	0.0001
		*θ*	0.0298	0.0024	0.0101	0.0018
	100	*α*	0.0112	0.0012	0.0110	0.0011
		*β*	0.0020	0.0001	0.0016	0.0001
		*θ*	0.0163	0.0029	0.0128	0.0016

**Table 5:** MSEs and biases for MLEs and bayes estimators of *α* = 2.5, *θ* = 1.5, *β* = 0.5.

			MLE		Bayes (SEL)	
Scheme	n	Parameters	Bias	MSE	Bias	MSE
1	50	*α*	0.15721	0.04950	0.15172	0.04604
		*β*	0.03752	0.00819	0.07588	0.01152
		*θ*	0.21066	0.10098	0.20710	0.14268
	100	*α*	0.15773	0.02491	0.15381	0.02366
		*β*	0.04296	0.01355	0.03700	0.00137
		*θ*	0.22702	0.06500	0.21497	0.06237
2	50	*α*	0.03156	0.00249	0.03074	0.00236
		*β*	0.00725	0.00032	0.00712	0.00015
		*θ*	0.03770	0.00381	0.03481	0.00296
	100	*α*	0.01567	0.00246	0.01550	0.00240
		*β*	0.00443	0.00036	0.00353	0.00027
		*θ*	0.01903	0.00388	0.01455	0.00212
3	50	*α*	0.03153	0.00249	0.03074	0.00236
		*β*	0.00745	0.00033	0.00782	0.00015
		*θ*	0.03804	0.00388	0.02881	0.00296
	100	*α*	0.01563	0.00245	0.01551	0.00241
		*β*	0.00441	0.00037	0.00347	0.00026
		*θ*	0.02095	0.00473	0.01443	0.00208

The corresponding 95% prediction confidence intervals (PI) for the non-observed order statistics $Y_{s:r_{j}}$ are reported in Tables (6–8). In Table 6 censoring scheme 1 is used and we can find the interval lengths which will lead us to the best predicted interval estimation for the unobserved $Y_{s:r_{j}}$. Since the BUP has shorter interval length than the BP we conclude that BUP is preferable to predict $Y_{s:r_{j}}$. For other censoring schemes we can obtain different results as shown in Tables 7 and 8, where the best interval estimation is by using BP.

**Table 6:** Point and interval prediction of the unobserved order statistics $Y_{s:r_{j}},\;1\leq i\leq m$ for scheme 1, with *α* = 0.5, *β* = 0.5, *θ* = 0.5.

		BUP		BP	
n(m)	*Y*_*i*:*m*:*n*_	Predicted *Y*_*i*:*m*:*n*_	95% CI	Predicted *Y*_*i*:*m*:*n*_	95% CI
50 (5)	*Y*_1:1:50_	0.3523	(0.2861, 0.3847)	0.2254	(0.0789, 0.3029)
	*Y*_2:1:50_	0.3953	(0.2966, 0.4003)	0.2567	(0.1082, 0.6369)
	*Y*_3:1:50_	0.4009	(0.3259, 0.4446)	0.3697	(0.1938, 0.6827)
	*Y*_4:1:50_	0.4235	(0.3520, 0.4854)	0.3523	(0.2819, 0.7097)
	*Y*_5:1:50_	0.4384	(0.3784, 0.5239)	0.4189	(0.3655, 0.7588)
100 (10)	*Y*_1:1:100_	0.6453	(0.5017, 0.6583)	0.4808	(0.3256, 0.8083)
	*Y*_2:1:100_	0.6585	(0.5337, 0.6895)	0.4561	(0.3798, 0.8207)
	*Y*_3:1:100_	0.6717	(0.5601, 0.6937)	0.4688	(0.3959, 0 .8476)
	*Y*_4:1:100_	0.7209	(0.5853, 0.7656)	0.5532	(0.4093, 0.8607)
	*Y*_5:1:100_	0.7406	(0.6193, 0.8080)	0.5824	(0.4492, 0.8842)
	*Y*_6:1:100_	0.7984	(0.6425, 0.8617)	0.6239	(0.4679, 0.9023)
	*Y*_7:1:100_	0.8249	(0.6895, 0.9026)	0.6711	(0.5504, 0.9235)
	*Y*_8:1:100_	0.8611	(0.7264, 0.9217)	0.7431	(0.5862, 0.9693)
	*Y*_9:1:100_	0.9044	(0.7637, 0.9791)	0.8339	(0.6132, 0.9970)
	*Y*_10:1:100_	0.9859	(0.8253, 1.0965)	0.9332	(0.7298, 1.0190)

**Table 7:** Point and interval prediction of the unobserved order statistics $Y_{s:r_{j}},\;1\leq i\leq m$ for scheme 2, with *α* = 0.5, *β* = 0.5, *θ* = 0.5.

		BUP		BP	
n(m)	*Y*_*i*:*m*:*n*_	Predicted *Y*_*i*:*m*:*n*_	95% CI	Predicted *Y*_*i*:*m*:*n*_	95% CI
50 (5)	*Y*_1:1:50_	0.4349	(0.3058, 0.5158)	0.3852	(0.2702, 0.4047)
	*Y*_2:1:50_	0.5038	(0.4204, 0.5917)	0.4173	(0.2966, 0.4203)
	*Y*_3:1:50_	0.5581	(0.4577, 0.61932)	0.4309	(0.3279, 0.4576)
	*Y*_4:1:50_	0.5842	(0.4900, 0.6271)	0.4930	(0.3520, 0.5034)
	*Y*_5:1:50_	0.6148	(0.5181, 0.6821)	0.5381	(0.3784, 0.5596)
100 (10)	*Y*_1:1:100_	0.7234	(0.6190, 0.6260)	0.6859	(0.5017, 0.6983)
	*Y*_2:1:100_	0.7302	(0.5848, 0.7227)	0.7073	(0.5315, 0.7192)
	*Y*_3:1:100_	0.7686	(0.6282, 0.8058)	0.7295	(0.5692, 0.7301)
	*Y*_4:1:100_	0.8047	(0.7012, 0.9915)	0.7690	(0.5793, 0.7903)
	*Y*_5:1:100_	0.8212	(0.7683, 1.0960)	0.8064	(0.6486, 0.8463)
	*Y*_6:1:100_	0.8587	(0.7286, 1.1938)	0.8390	(0.6943, 0.8901)
	*Y*_7:1:100_	0.9091	(0.7655, 1.2074)	0.8973	(0.7386, 0.9315)
	*Y*_8:1:100_	0.9583	(0.7816, 1.2432)	0.9147	(0.8268, 1.0163)
	*Y*_9:1:100_	0.9970	(0.8525, 1.3628)	0.9736	(0.9192, 1.0986)
	*Y*_10:1:100_	1.1313	(0.9713, 1.3901)	0.9984	(0.9328, 1.1275)

**Table 8:** Point and interval prediction of the unobserved order statistics $Y_{s:r_{j}},\;1\leq i\leq m$ for scheme 3, with *α* = 0.5, *β* = 0.5, *θ* = 0.5.

		BUP		BP	
n(m)	*Y*_*i*:*m*:*n*_	Predicted *Y*_*i*:*m*:*n*_	95% CI	Predicted *Y*_*i*:*m*:*n*_	95% CI
50 (5)	*Y*_1:1:50_	0.5049	(0.2637, 0.5839)	0.4149	(0.2567, 0.4983)
	*Y*_2:1:50_	0.5372	(0.3591, 0.5711)	0.4890	(0.3733, 0.5191)
	*Y*_3:1:50_	0.5602	(0.4919, 0.6028)	0.5290	(0.4381, 0.5890)
	*Y*_4:1:50_	0.6137	(0.5280, 0.6954)	0.6049	(0.4801, 0.6479)
	*Y*_5:1:50_	0.6352	(0.5705, 0.7041)	0.6281	(0.5276, 0.7001)
100 (10)	*Y*_1:1:100_	0.7132	(0.5601, 0.7237)	0.7092	(0.5572, 0.7383)
	*Y*_2:1:100_	0.7489	(0.5853, 0.7656)	0.7398	(0.5715, 0.7609)
	*Y*_3:1:100_	0.7794	(0.6193, 0.8080)	0.7692	(0.6092, 0.8015)
	*Y*_4:1:100_	0.8083	(0.6425, 0.8617)	0.8010	(0.6394, 0.83903)
	*Y*_5:1:100_	0.8361	(0.7295, 0.9026)	0.8201	(0.6895, 0.8563)
	*Y*_6:1:100_	0.9301	(0.8393, 0.9784)	0.9098	(0.7543, 0.9215)
	*Y*_7:1:100_	1.0184	(0.8994, 1.1297)	0.9375	(0.8469, 0.9615)
	*Y*_8:1:100_	1.0679	(0.9582, 1.1614)	0.9673	(0.8968, 0.9934)
	*Y*_9:1:100_	1.1068	(0.9971, 1.2074)	1.0731	(0.9284, 1.11847)
	*Y*_10:1:100_	1.1829	(1.0356, 1.2406)	1.1092	(0.9628, 1.1589)

## 4. Discussion

The MLE based on EM algorithm and Bayes estimates under SEL function using MCMC method are obtained and displayed in Table 2. Also, the approximate asymptotic confidence intervals and the credible intervals are computed and tabulated in Table 2. From Table 2, we notice that the MLE and the Bayes estimates are co-inside and the Bayes credible intervals have shorter length than the approximate confidence intervals. The analysis of the proposed data set demonstrates the applicability and the importance of the proposed model.

To check the validity of Marshall-Olkin Pareto (MOP) distribution to fit this data set, we use the Kolmogorov–Smirnov (K–S) test is applied. The K–S distance and its respective p-value are computed to be K − S = 0.1304 and p-value = 0.8441, respectively. Therefore, it is quite reasonable to indicate that the MOP distribution is fitting this data well.

**Fig 3:**
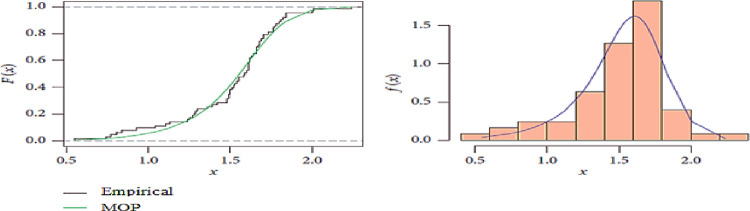
Plot of the max K–S distance between two empirical CDF, and histogram of MOP distribution for data set.

The relative histograms and fit of MOP distribution of data sets are discussed in Fig 3, with the plot of the max distance between the two empirical CDF curves for MOP distribution. Moreover, it indicates that MOP distribution can be fitted to the data set.

**Fig 4:**
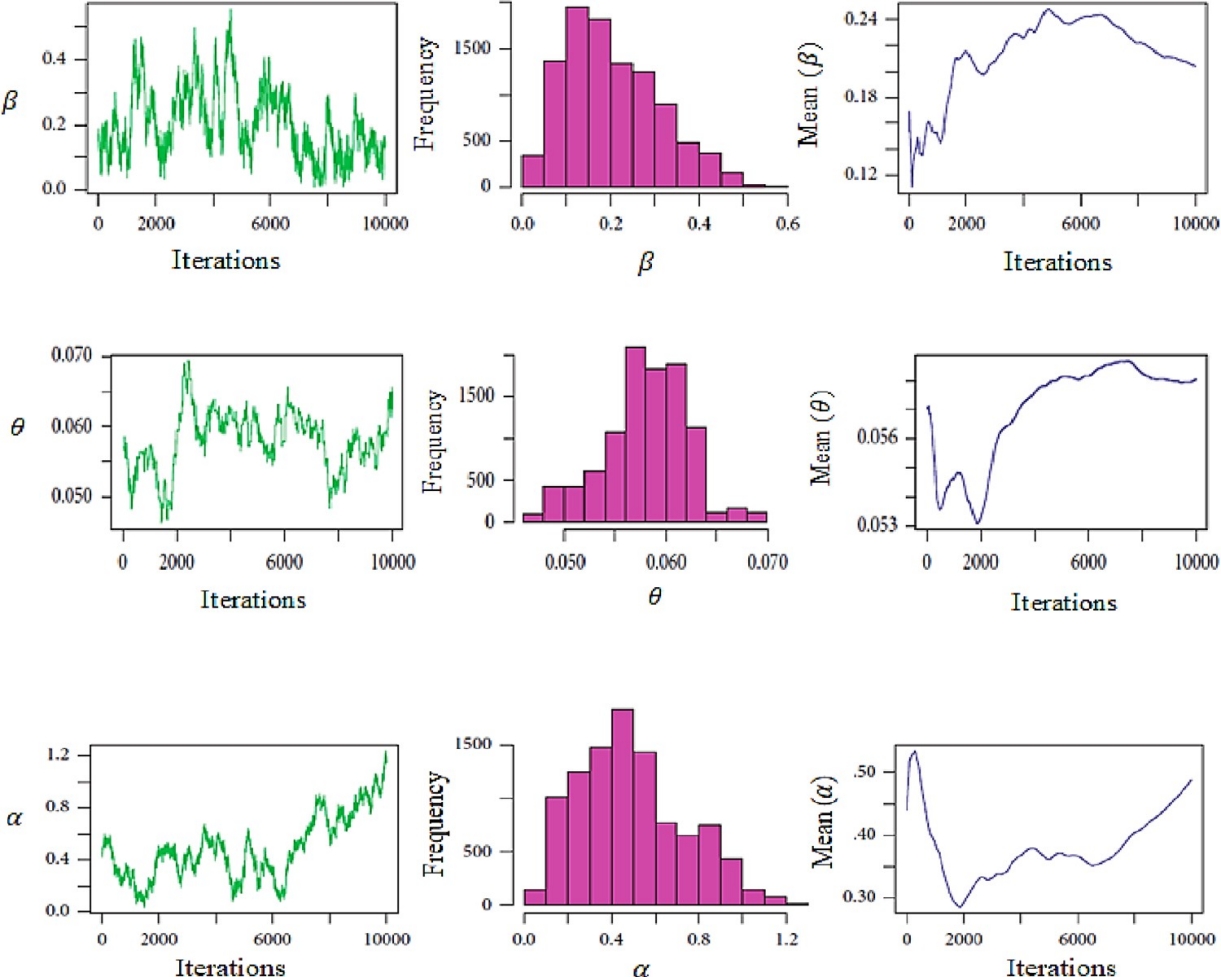
MCMC plots based on Type-II censored sample of MOP distribution for data set.

In addition to histogram plots, approximate marginal posterior density, and MCMC convergence of ***α***, ***β***, **and *θ*** are represented for data set in Fig 4.

Suppose the life test ended when the 15th observation is observed, i.e., we observe a Type-II censored sample with ***n* = 20** and ***m* = 18**. Based on the prediction methods presented in Section 2, we computed the point predictors, these results are presented in Table 3. It is clearly evident that the point predictors are very close to the true values for different Schemes. Moreover, the Predicted value under BP is the better than Predicted value under BUP for different Schemes.

In simulation algorithm, Monte Carlo experiments were carried out under the following data generated from MOP distribution, where x is distributed as MOP distribution for different parameters ***Ω*** = (***α*, *β*, *θ***) and the initial values of the parameters are as follows:

**Case 1: *α* = 2, *θ* = 1.5, *β* = 0.5**
**Case 2: *α* = 2.5, *θ* = 1.5, *β* = 0.5**
**Case 3: *α* = 0.5, *θ* = 0.5, *β* = 0.5**

In Tables 4 and 5, we present the MLE for ***α***, ***β***, **and *θ*** as well as the Bayes estimates of ***α***, ***β***, **and *θ***, under the loss functions. Numerical results of Bayes estimate for ***α***, ***β***, **and *θ***, and their corresponding Bias and MSE values are computed in case 1,2 respectively, for different sample size (***n*)** under different schemes. We observe that the Bayes estimates perform better than MLE in terms of Bias and MSE values. For fixed ***n***, when the number of failures increase the Bias MSE decrease in all cases and for different estimators. Comparing the three censoring schemes, it is observed that in most of the case 1and 2, Scheme 3 perform better than schemes 1 and 2 in terms of Bias and MSE values.

Graphically, illustrate the MSE values for different estimates in all cases with different schemes are shown in Figs 1 and 4 respectively. In this Figures, is concerned with the MSE values for different estimates in all cases under different schemes, it is clear that the scheme 1has the largest MSE values, followed by scheme 2, finally scheme 3. Moreover, this Figures confirms that scheme 3 is the best schemes, especially when ***n*** increases.

In Tables 6–8 we have reported the lifetime of higher order units increases as n increases and the length of predictive interval become wider as well for case 3 under different schemes. We have reported best unbiased predictor (BUP) and Bayes predictive estimates (BP) for both set of parameters, and predictive interval for both set of parameters are reported based on classical approach. Here the lifetime of higher order units become larger and length of predictive interval becomes wider.

In Tables 6–8 we have reported the lifetime of higher order units increases as n increases and the length of predictive interval become wider as well for case 3 under different schemes. We have reported best unbiased predictor (BUP) and Bayes predictive estimates (BP) for both set of parameters, and predictive interval for both set of parameters are reported based on classical approach. Here the lifetime of higher order units become larger and length of predictive interval becomes wider.

From Table 6 we present BUP and BP prediction estimates along with pivotal and CI prediction intervals for case 3 based on Scheme 1. Table 7 we present BUP and BP prediction estimates along with pivotal and CI prediction intervals for case 3 based on Scheme 2. While in Table 8 addresses BUP and BP prediction estimates along with pivotal and CI prediction intervals for case 3 based on Scheme 3. Moreover, that the predicted values for the missing ***i***^***th***^ order statistics ***Y***_***i*:*m*:*n***_ based observed sample of size *m* with censoring schemes for all schemes described above under the loss function for fixed choice of case 3. Based on MCMC samples (**Ω**_***i***_; ***i*** = **1,2,…*M***), ***M* = 10,000** the Bayes point prediction for the missing order statistics ***Y***_***i*:*m*:*n***_ in censoring stage are computed under the loss function. The 95% lower bound L and upper bound U of prediction interval for the ***i***^***th***^ order statistics ***Y***_***i*:*m*:*n***_ are also computed.

In general BP estimates are slightly smaller than BUP and Pivotal Interval are slightly larger than 95% CI prediction interval.

## 5. Conclusions

In this article, we used two prediction methods to predict the unobserved units under progressive Type-II censored sampling, the lifetimes followed Marshall-Olkin Pareto model. Point and Interval estimation of the unknown parameters of Marshall-Olkin Pareto distribution are obtained using classical (MLE) and non-classical (Bayes) estimation. Numerical analysis using EM algorithm was performed to find the numerical solution of the MLE, and the MCMC method was used for Bayesian calculations. A simulation study was conducted to assess the performance of these estimation methods. Based on the simulation results, we showed the advantage of using Bayesian method over the classical method of estimation. A real data example was used to determine the best prediction method; hence we observed the advantage of the Bayesian prediction method over the BUP method.

## Supporting information

S1 File (DOCX)

S1 Dataset (PNG)
