# Supplementary material for: Progressive censoring schemes for marshall-olkin pareto distribution with applications: Estimation and prediction
Source: PLoS One. 2022 Jul 27;17(7):e0270750. doi: 10.1371/journal.pone.0270750 (PMC9328570; doi:10.1371/journal.pone.0270750)
Supplement: S1 File — (DOCX) [file pone.0270750.s001.docx]

**Real Data**

We consider a progressively censored real data set from [1], it consists of the failure times of 20 mechanical components, see Table. 1

**Table 1.** Data set of failure times of 20 mechanical components

| 0.067 | 0.068 | 0.076 | 0.081 | 0.084 | 0.085 | 0.085 | 0.086 | 0.089 | 0.098 |
| --- | --- | --- | --- | --- | --- | --- | --- | --- | --- |
| 0.098 | 0.114 | 0.114 | 0.115 | 0.121 | 0.125 | 0.131 | 0.149 | 0.160 | 0.485 |

1. Murthy, D. N. P., Xie, M., and Jiang, R. (2004). Weibull models, Wiley series in probability and statistics, John Wiley and Sons
