# Supplementary figures and images for: Progressive censoring schemes for marshall-olkin pareto distribution with applications: Estimation and prediction
Source: PLoS One. 2022 Jul 27;17(7):e0270750. doi: 10.1371/journal.pone.0270750 (PMC9328570; doi:10.1371/journal.pone.0270750)

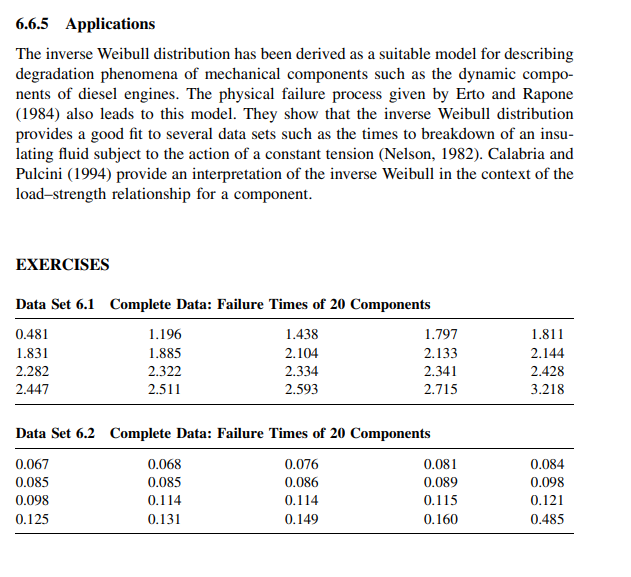

Supplement: S1 Dataset — (PNG) [file pone.0270750.s002.png]
